# Predicting carrying capacity of a large carnivore from prey densities: a new approach

**DOI:** 10.7717/peerj.15914

**Published:** 2023-11-22

**Authors:** Nilanjan Chatterjee, Indranil Mukhopadhyay, Parag Nigam, Bilal Habib

**Affiliations:** 1 Wildlife Institute of India, Dehradun, India; 2 Human Genetics Unit, Indian Statistical Institute, Kolkata, India

**Keywords:** Carnivore density, Scaling, Prey density, Tiger, Carrying capacity

## Abstract

**Background:**

Large carnivores play a crucial role in maintaining the balance of the ecosystem. Successful conservation initiatives have often led to a huge increase in predators which has often led to negative interactions with humans. Without the knowledge of the carrying capacity of the top predator, such decisions become challenging. Here, we have derived a new equation to estimate the carrying capacity of tigers based on the individual prey species density.

**Methods:**

We used tiger densities and respective prey densities of different protected areas. Relative prey abundance was used instead of absolute prey density as this could be a better surrogate of the prey preference. We used a regression approach to derive the species-wise equation. We have also scaled these coefficients accordingly to control the variation in the standard error (heteroscedasticity) of the tiger density. Furthermore, we have extended this regression equation for different species to different weight classes for more generalized application of the method.

**Results:**

The new equations performed considerably better compared to the earlier existing carrying capacity equations. Incorporating the species-wise approach in the equation also reflected the preference of the prey species for the tiger. This is the first carrying capacity equation where the individual prey densities are used to estimate the carnivore population density. The coefficient estimates of the model with the comparison with prey-predator power laws also reflect the differential effect of tigers on different prey species. The carrying capacity estimates will aid in a better understanding of the predator-prey interaction and will advance better management of the top predator.

## Introduction

Mammalian carnivores are susceptible to habitat fragmentation, disturbance, and exploitation by humans, and so are severely threatened with extinction and are declining globally (Ginsberg, 2001). Large carnivores, being at the top of the food chain, have a pivotal role in maintaining the functional integrity of diverse ecosystems (Ripple et al., 2014). They influence and can regulate prey by imposing top-down control and mesopredators through interference competition (Hoeks et al., 2020). In the last 150 years, tigers *Panthera tigris* have lost around 93% of their global historical range through habitat loss and fragmentation, hunting and depletion of wild prey species (Sanderson et al., 2006). The situation is almost the same in the Indian sub-continent where tigers occur in only 11% of their historical range, yet this region still harbours around 60% of the global wild tiger population (World Wildlife Fund, 2016). In 2010, the governments of 13 tiger range countries adopted the St. Petersburg Declaration to double the population of wild tigers by 2022. The crux of this goal depends on recovering potential habitats and enhancing prey populations. However, doubling tiger numbers by 2022 will depend on efforts toward restoring interactions between species at each trophic level from local to global scales. Understanding local scale interactions are critical to advance towards the global goal and holds the key for recovering large carnivore as well as the whole trophic system. Instead of adequate knowledge about local-level interactions, our ability to instigate recovery at a global scale will be limited.

Three decades of conservation success in India have resulted in rapid growth in some tiger populations (Jhala et al., 2021). The rapid increase in tiger numbers is making these tigers venture into human-dominated landscapes, increasing conflict with livestock and humans, which may lead to the development of negative attitudes and declining tolerance among local people (Habib et al., 2015). Under the scenario of increasing tiger populations at specific sites, interventions like conservation translocations seem to be an effective measure in securing other slow recovering sites to maintain population viability and achieve the global tiger recovery goal. Before such management and policy decisions, it is of fundamental importance to estimate the carrying capacity that can be sustained by the reserve, so as not to create the situation of overabundance at local scales leading to disruption of the ecological balance and declining tolerance of neighbouring people. With road development interrupting corridor connectivity (Carter et al., 2020), maintaining meta-population information on reserve level population sizes, colonisation and local extinction would benefit strategizing recovery programmes aiming at local populations to achieve global goals.

Most carnivores are limited by food resources, their density can be established as a function of prey availability (Fuller & Sievert, 2001). The rate of food intake of a predator can be expressed as a function of available food density; this is known as a functional response (Holling, 1959). Recent ecological literature considers more complexity in such cases, as the predator density is not only determined by prey density but also determines the prey density (Abrams & Ginzburg, 2000). Arditi & Ginzburg (1989) proposed an alternative form of prey dependence as ratio-dependence in which the response only depends on the ratio of prey population size to predator population size, not on the absolute number of the species. Prey-predator interactions are dynamic and evolve to adapt to each circumstance. If this dynamic equilibrium is lost, either the prey or predator will become extinct.

Carbone & Gittleman (2002) developed an equation based on the prey biomass and predator weight for a large set of carnivores, using a limited number of studies from a few sites not representing an overall range of prey species consumed by a carnivore. As all the available prey are not predated upon proportionately, this equation produced a very coarse relationship. Subsequently, Karanth et al. (2004) also developed an equation focused on the tiger and their ungulate prey based on nine sites mostly limited within the well-known tiger reserves and therefore not capturing the entire variation in tiger habitat and associated species. The variation in prey density throughout the tiger reserves was not represented properly in the equation with their low sample size and large error bars were present reducing the value of this equation for management purposes.

The interplay between density and biomass and the role of relative availability of prey remains poorly understood. (Miquelle et al., 2010) tried to build an equation based on prey biomass and tiger density from different sites of the tiger range. They used the data from 13 sites throughout the tiger range with varied prey species community and found a curvilinear relation between tiger density and prey biomass.

As predicted by Ruxton (2005), if a predator increases its search rate, the encounter rate with prey increases with prey density at a slower rate than expected from a directly proportional relationship. Under such circumstances using density or biomass may give skewed results while estimating the carrying capacity of predator species. There is potential to develop predictive equations relating prey densities to that of tigers, however, more work needs to be done to make these useful and applicable in a broader perspective.

In this study, we used data from 23 sites in India across the tiger distribution range holding more than 60% of the global tiger population. Using the data on the estimated population density of different prey species, we multiplied it by the prey weight and calculated the total prey biomass. Dividing the different prey species’ weight by the total prey biomass, we subsequently converted the absolute prey density into relative prey density. Relative density was used despite actual density as it can function as a better surrogate for the prey-preference. The relative prey biomass was then regressed with the estimated tiger density to test the prey-predator relationship. We used multiple regression equations and information about relative prey density to predict tiger density across the sites in India. We compared the results of our modelled equation with existing scaling equations. We believe that with a better understanding of predator carrying capacity, based on relative prey density, more informed conservation decisions can be made about managing human-tiger conflict and conservation translocations in India and other tiger range countries aiming at achieving global conservation goals.

## Materials and Methods

Carnivores are constrained by the available prey biomass. Tigers, being the largest felids, have a large spectrum of prey animals ranging from a 10 kg langur *Semnopithecus sp.* to >600 kg gaur *Bos gaurus* (Karanth et al., 2004). Earlier field studies found an estimate of 2,868 kg and 3,389 kg of prey required annually for an adult female and male tiger respectively (Støen & Wegge, 1996). (Karanth et al., 2004) mentioned 50 ungulates per year to sustain an adult tiger but the required biomass (Støen & Wegge, 1996) can be obtained from eight gaurs (average 400 kg) or 20 Sambar/Nilgai (average 150 kg) or 60 Chital/Wild-Pig (average 50 kg). We feel these numbers of prey are underestimates given the presence of kleptoparasites and the decomposition rate given the climatic condition of the Indian subcontinent. Hence, in this equation, we used relative prey density biomass as a surrogate for prey preference and rate of predation in our equation to control the heterogeneity in the prey biomass. This approach would improve the scaling equation by taking into account the species ecology and providing robust estimates compared to utilizing the earlier equations using prey density.

### Database generation

We reviewed the literature on predator–prey equations. Data was collected from one source to control the various sampling approach and analytical methods. Tiger and prey densities were obtained from the all India Tiger Report 2014 (Jhala, Qureshi & Gopal, 2015). We have considered density estimates of 23 sites where both tiger and prey density was available. As we have collected the data mostly from a single study, the dataset is not constrained by different sampling techniques, objectives, and analytical methods. Dataset from these sites was used to model the scaling equation. As the 23 sites were spread across a variety of habitats throughout India across the tiger distribution range, where tiger populations varied in sex ratio and age structure, the bias arising from age/sex-related preference for species and age-sex class was avoided. Prey densities were converted into biomass with 3/4 of adult female biomass (Schaller, 1972; Hayward, O’Brien & Kerley, 2007). Biomass was used rather than ungulate density to account for the weight range of prey killed. We used proportions of the biomass of each prey species with the log-transformed biomass of the prey species. The proportional biomass was used for the equations as it can serve as a robust indicator of site-wise prey preference, as we only used prey species from the preferred weight range of tiger. We pooled the biomass of species in similar weight classes to assess the relationship between tiger density with different weight range classes.

### Analytical approach

We regressed log-transformed predator density with the product of log-transformed biomass of each prey species and the proportion of the prey biomass. The tiger density was calculated per 100 sq. km while the prey density was calculated per 1 sq. km. We used all the major prey species available but excluded barking deer (*Muntiacus muntjak*) and grey langur (*Semnopithecus* sp.) as studies (Hayward, Jedrzejewski & Jedrzejewska, 2012) have found tigers prefer a prey body mass of 60–250 kg and hence, they are not the most preferred prey of Tigers. For the preferred weight range equation, prey species were classified into four categories, low (0–25 kg), medium (25–80 kg), high (80–250 kg) and very high (>250 kg). Low-weight range prey species were not taken into account because of their minimal contribution to the tiger diet. The log-transformed tiger density was regressed with the different weight class categories to obtain the predictive weight range equation. We also investigated heteroskedasticity in individual prey species and removed that using square-root transformation. Furthermore, we tested the equation to estimate tiger densities in areas with reintroduced populations. Codes to perform the analysis can be found in the File S1. Analysis was carried out in lmtest (Zeileis & Hothorn, 2002) and sandwich (Zeileis, Köll & Graham, 2020) package in R 3.6 (R Core Team, 2019).

### Functional response and predator–prey power law

We assumed a Type II or Type III functional response (Holling, 1959) for the relationship between prey biomass and tiger density. Type I functional responses were not considered in this case because an increase in prey density cannot indefinitely lead to increased predation as the predators are limited by other life-history traits and capacity to process food intake. They are also territorial and defend an area as their home range. The Type II functional response has also been reported from wolf-caribou (Dale, Adams & Bowyer, 1994) and wolf-moose (Hayes & Harestad, 2000) from different areas. (Karanth et al., 2004) and Miquelle et al. (2010) also assumed a Type II response for building their tiger-prey equation. We did not use the Michaelis–Menten function (y = a.e^*b*/*x*^) (Michaelis & Menten, 1913) for Type-II functional response though we use the proportion of species biomass in each area raised to the species biomass for building both the prey species and weight range equation.

We also fitted our data from the predator–prey power law equation given in Hatton et al. (2015). The power-law equations are of the form y = cx^k^ (here, *y* is the biomass of predator, *x* is the biomass of prey, *c* is known as the coefficient and k is known as the scaling exponent). *k* >1 indicates top-down control of the predator species on the prey species, *k* = 1 means an equilibrium system and constant transfer of biomass to each successive trophic level whereas *k* <1 means bottom-heavy relation between trophic levels.

Hatton et al. (2015) tried to find the link between biomass production and predator–prey interactions. The study found a similar exponent throughout the different ecosystem and body-mass allometries. We also checked the exponent with our derived equation. We compared our estimated densities with previously available equations available for tigers to compare the predictive accuracy of our equation. We estimated tiger densities of different sites using prey density from this study following the equations given by Carbone & Gittleman (2002), Karanth et al. (2004), Hayward, O’Brien & Kerley (2007) and Miquelle et al. (2010). Though Hayward, O’Brien & Kerley (2007) built an equation to estimate the carrying capacity of African carnivores, the equation has been used to predict the carrying capacity of tigers (Roy et al., 2016). We calculated the correlation value of the observed tiger density with the fitted tiger density of each equation. We also tested the estimated correlation value against the hypothesis of no correlation between the observed and predicted tiger densities.

## Results

### Tiger-prey scaling

The tiger density at each site was found to be significantly related (*r*^2^ = 0.82, *F*_6,17_ = 18.58, *p* < 0.001) to the biomass of the prey species and/or significantly related to the prey weight range (*r*^2^ = 0.452, *F*_3,19_ = 7.05, *p* = 0.002). We considered these equations for predictions of the population density and carrying capacity of a tiger at the sites. We found heteroskedasticity in the chital variable in the prey-species equation (Fig. S1). It was further removed from the equation by the square-root transformation mentioned. No heteroskedasticity was detected in the weight equation model.

The equation derived in this study is given below.

#### Prey-species equation

Log (tiger density)*k = −1.549*k + 1.142*k*(proportion of chital biomass) *log (chital biomass) + 0.566*k*(proportion of sambar biomass) *log (sambar biomass) + 0.698*k*(proportion of gaur biomass) *log (gaur biomass) + 0.741*k*(proportion of wild pig biomass) *log (wild pig biomass) + 0.404*k*(proportion of nilgai biomass) *log (nilgai biomass) where, k = sqrt((proportion of chital biomass) *log (chital biomass)+1).

#### Weight equation

Log (tiger density) = −1.697+ 1.135*(proportion of sp_bio_mid) *log (sp_bio_mid) + 0.552*(proportion of sp_bio_high) *log (sp_bio_high) + 0.717*(proportion of sp_bio_very high) *log (sp_bio_very high) where,

sp_bio_mid stands for medium weight species biomass (25–80 kg)

sp_bio_high stands for high weight species biomass (80–250 kg) and

sp_bio_very high stands for high weight species biomass (>250 kg).

### Comparison with other works

**Figure 1:**
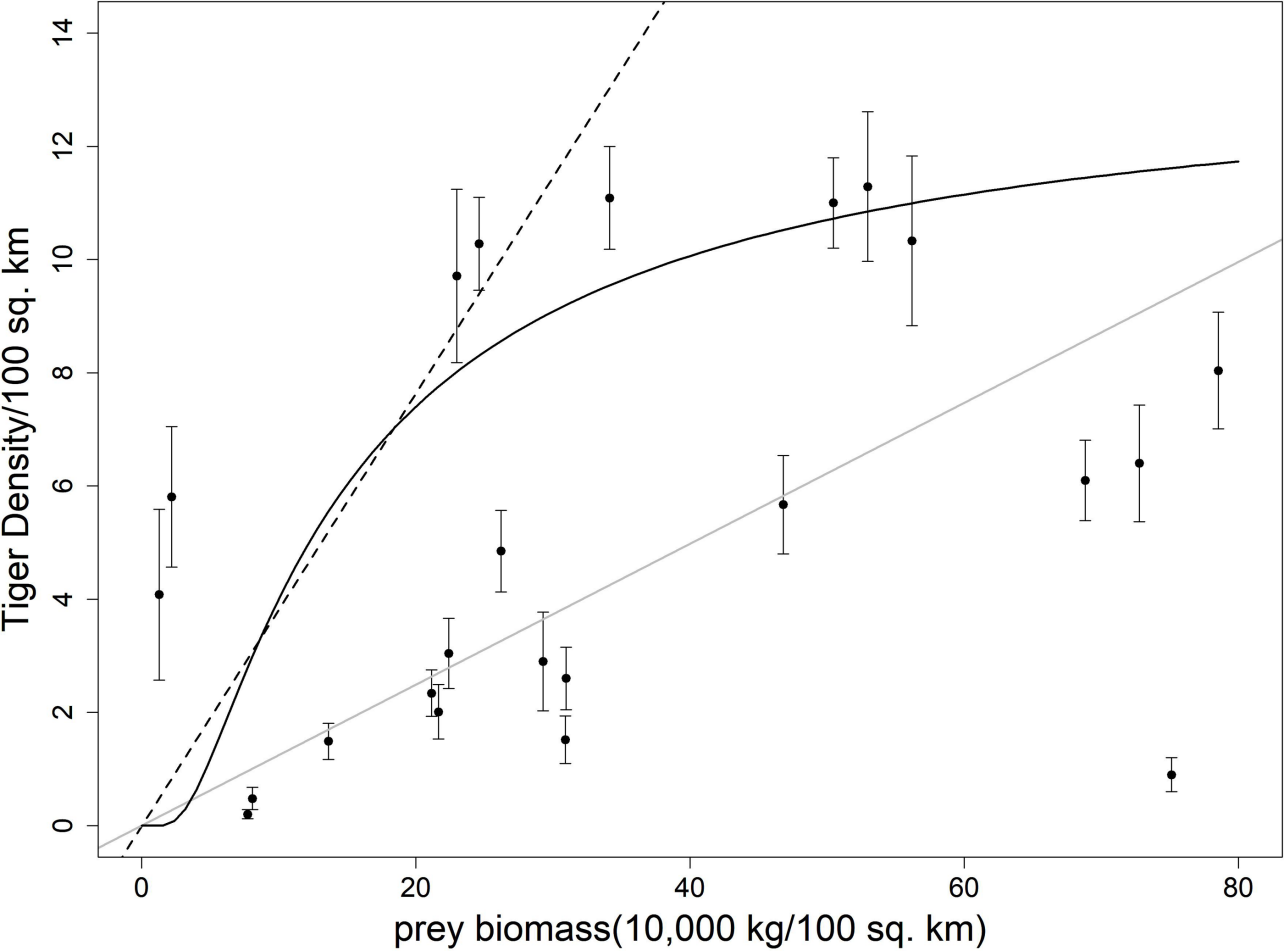
Plot showing ungulate prey biomass and tiger density with respective standard error of 23 sites across India. The dotted line represents the equation from Carbone & Gittleman (2002), the grey solid line represents the fitted line to the scatterplot, the black solid line represents the equation given in Miquelle et al. (2010).

**Figure 2:**
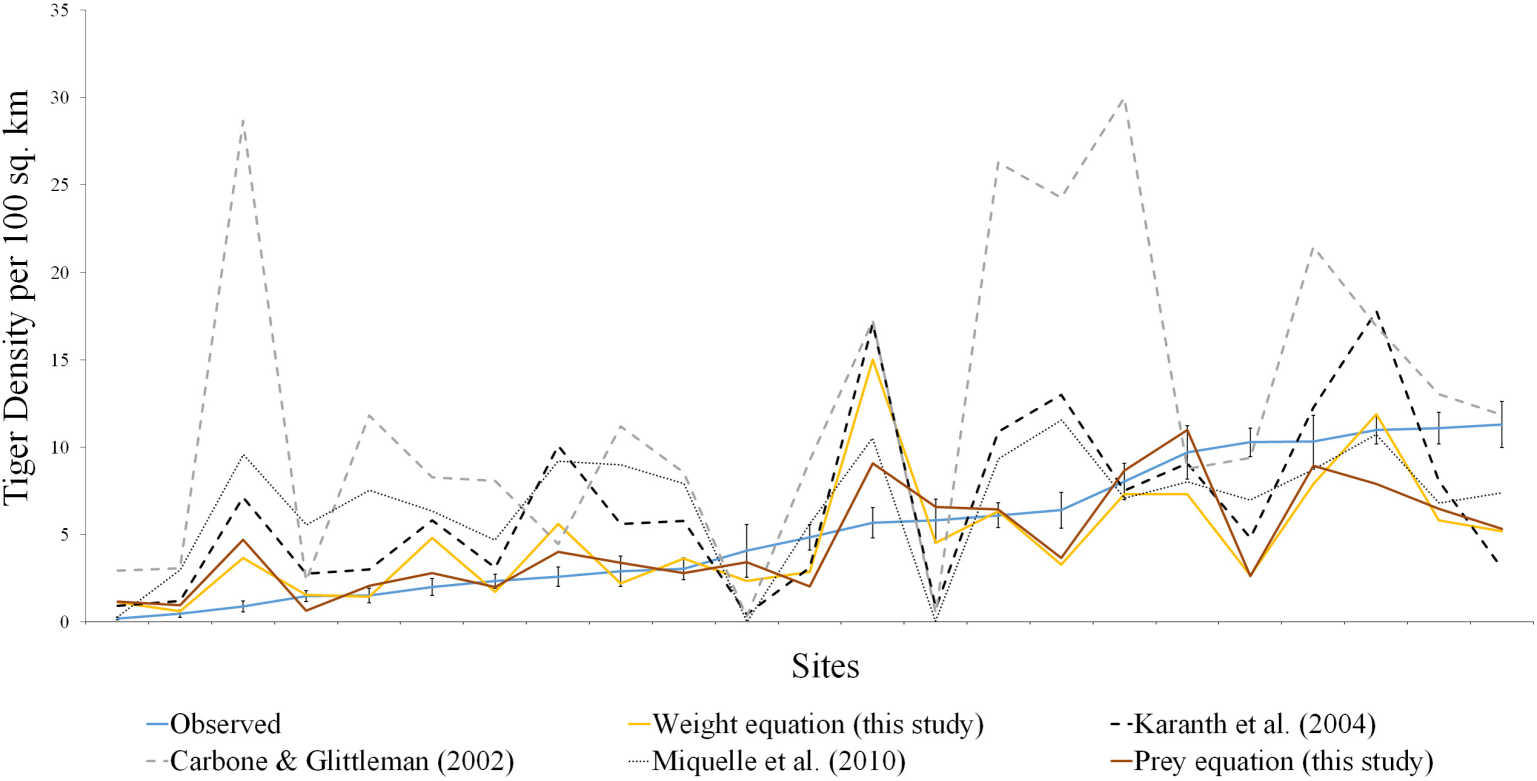
Comparison of estimated tiger densities with estimates of this study and previously published carrying capacity equations. The brown line represents the prey species equation, yellow line denotes the weight equation, blue line is actual tiger density with standard error, the dotted line represents the equation from Carbone & Gittleman (2002), Karanth et al. (2004), Miquelle et al. (2010).

Among the previously published studies on the carrying capacity equation, Karanth et al. (2004) did not report any goodness of fit measure. Miquelle et al. (2010) reported a higher r-square value but the sample size (*n* = 13) was less in his case. We tried to fit the equation derived in Carbone & Gittleman (2002), Karanth et al. (2004) and Miquelle et al. (2010) to our dataset. All the equations seemed to be overestimating the tiger density (Table 1, Figs. 1 and 2).

**Table 1:** Observed tiger density & standard error (SE) per 100 sq.km along with predicted tiger densities of each site based on (Karanth et al., 2004; Carbone & Gittleman, 2002; Miquelle et al., 2010) and this study.

**Sites**	**Observed** **tiger density (SE)**	Karanth et al. (2004)	**Carbone & Gittleman (2002)**	**Miquelle et al. (2010)**	**Prey-species equation (this study)**	**Weight equation (this study)**
Anshi	0.20 (0.08)	0.93	2.95	0.30	1.179	1.13
Simlipal	0.48 (0.20)	1.20	3.08	2.98	0.963	0.63
Pakke	0.90 (0.30)	7.14	28.69	9.57	4.725	3.67
Valmiki	1.49 (0.32)	2.78	2.34	5.55	0.663	1.55
Satpura	1.52 (0.42)	3.01	11.80	7.55	2.083	1.45
Kanha (buffer)	2.01 (0.48)	5.81	8.27	6.36	2.801	4.83
Bhadra	2.34 (0.41)	3.12	8.08	4.71	2.016	1.71
Pilibhit	2.60 (0.55)	10.10	4.48	9.20	4.017	5.62
Rajaji	2.90 (0.87)	5.62	11.18	8.99	3.403	2.20
Pench MH	3.04 (0.62)	5.78	8.55	7.90	2.788	3.63
Sundarban (2010)	4.08 (1.51)	0.44	0.29	0.00	3.415	2.36
Tadoba	4.85 (0.72)	3.11	9.26	5.59	2.029	2.88
Pench MP	5.67 (0.87)	17.09	17.22	10.52	9.075	15.02
Sundarban (2012)	5.81 (1.24)	0.79	0.49	0.05	6.576	4.52
Kanha (core)	6.10 (0.71)	10.88	26.29	9.33	6.432	6.34
Ranthambore	6.40 (1.03)	12.98	24.28	11.56	3.653	3.28
Ramnagar	9.71 (1.53)	9.08	8.77	8.01	8.662	7.30
Bandipur	10.28 (0.82)	4.82	9.39	6.98	10.996	2.67
Wayanad	10.33 (1.50)	12.26	21.46	8.72	2.63	7.90
Corbett	11.00 (0.80)	17.77	16.92	10.72	8.958	11.90
Nagarhole	11.09 (0.91)	8.09	13.03	6.82	7.914	5.83
BRT	11.29 (1.32)	3.10	11.85	7.38	6.492	5.21
Mudumalai	8.04 (1.03)	7.51	30.00	7.10	5.337	7.31
R^2^ value		0.47	0.28	0.23	0.68	0.48
*p*-value		0.04	0.25	0.29	0.001	0.04
*t*-test *p*-value		0.93	0.99	0.97	0.83	0.14

The coefficient for tiger density and ungulate density (0.131) (SE 0.02) (r-square 0.70, *p* < 0.001), which was significantly different from the value of A as 0.247 (95% CI [0.181–0.336]) reported in (Karanth et al., 2004). The coefficient for the Carbone & Gittleman (2002) using tiger weight of 180 kg was 0.381 but when it was fitted to our data it revealed a coefficient of 0.125 (r-square 0.63, *p* < 0.001). Though the coefficient was within the 95% confidence interval of (0.09−2.45), the interval was quite large for scaling purposes.

Hatton et al. (2015) found the scaling exponent of predator and prey biomass is very close to 0.75. Our overall equation fit was 0.33 (95% CI [−0.09 to −0.57], r-square 0.07), significantly different from 0.75 but when we compared it with the coefficient of individual prey and weight class then most of the coefficients had an overlap with 0.75. Chital in the prey-species equation and medium weight class had a coefficient greater than 1 in the power-law equation.

## Discussion

Finding the relationship between predator and prey density is one of the great challenges in ecology. The outcome being multi-faceted, can be used for long-term conservation implications, better management and understanding predator behaviour in depth. Such macro-ecological studies are usually constrained by the availability of data at a large spatial scale and field/analytical approaches of different studies, but we negotiated these problems by incorporating the entire Indian range of the tiger and using data collected following a similar sampling protocol (Jhala, Qureshi & Gopal, 2015). Although there are debates regarding the data collection and reporting (Harihar et al., 2017; Qureshi, Gopal & Jhala, 2019) but as there are no other datasets available across the macroecological scale, we were obligated to use them for our analysis. There are several such equations (Carbone & Gittleman, 2002; Karanth et al., 2004; Miquelle et al., 2010) but most of them lack the robustness of several sampling sites or many studies reviewed. Such models have been rarely used to evaluate site-wise population density and carrying capacity for management interventions like conservation translocation or reintroduction. Moreover, all the earlier equations did not incorporate species level density and differential species preference information to calculate the carrying capacity. Apart from understanding carrying capacity, the equation of this study can also be used for the species-specific role and to identify changes due to the difference in prey diversities.

Carbone & Gittleman (2002) used only six populations of the tiger to derive the equation for all the carnivore species. Karanth et al. (2004) used 11 study locations for the study but used nine locations for analyses. Both these studies used the density of prey species to predict the density of predators. (Miquelle et al., 2010) used data from 13 sites to derive the equation for two subspecies *P. tigris tigris and P. tigris altaica*. Both Carbone & Gittleman (2002) and Miquelle et al. (2010) used different subspecies (*P. tigris tigris and P. tigris altaica*) inhabiting completely different habitats and climatic conditions to build their equation. Both these studies used prey biomass, whereas prey density was used by Karanth et al. (2004). Our study used data from 23 different sites of the same tiger subspecies covering all possible habitats and range of tiger and prey densities. With the integration of species-specific effects in this equation, it is much more generalizable to diverse prey community present across the sites compared to the earlier ones. We believe the weight equation is a generalized version of the individual species equation and can be used for other subspecies of tiger for carrying capacity estimation.

In comparison to the previous equations, our model is more conservative given our larger sample size and using proportional biomass as a surrogate to the prey preference. Our model predicts significantly well for the low and medium-density sites (Fig. 2; Table 1) but for high-density sites, it underestimates predator density. The regulation imposed in the high-density sites is through social behaviour rather than competition (Støen & Wegge, 1996; Miquelle et al., 2010). With low habitat productivity and low prey density, large carnivores have to spend more time searching for food resources to meet their energy requirements (Valeix et al., 2010; Powell & Boitani, 2012). In such situations, their survival, as well as reproduction, must be reduced, while sites with a higher biomass of prey species offer easy access to prey for tigers. This reduces the amount of energy and time spent on hunting. This, in turn, may help them to concentrate on other activities that lead to higher survival rates, breeding success and better scope for reproduction, which leads to a higher density of carnivores in the areas with high prey biomass. This study focused more on the response of predators with different prey densities, and the absence of social interactions in the predator density estimates resulted in the departure of the expected densities from those observed. Given the current scenario of habitat loss and prey depletion, it is of fundamental importance to gain a better understanding of the predator density with different prey species and their weight ranges.

As we used a high number of correlated variables in our model building, there is a high chance of overfitting the dataset. But as the sample size was not very high, we validated the equation to estimate tiger densities of reintroduced sites. After the local extermination of tigers from the Panna tiger reserve in 2009, tigers were reintroduced from adjoining tiger reserves. Tiger density from the reserve calculated in 2012–2013 was 1.54 and 1.59/100 sq. km (Ramesh et al., 2013) which was very close to our estimate of 1.48 tigers/100 sq. km. In subsequent years when the tiger number increased and exceeded the estimated carrying capacity of 30, tigers dispersed from the reserve (Krishnamurthy et al., 2016). Sariska Tiger Reserve also lost all its Tigers in 2005 and, in 2008, tigers were reintroduced from Ranthambore Tiger Reserve (Gopal et al., 2010). The current tiger density was reported as 1.64/100 sq. km (Jhala, Qureshi & Gopal, 2015) whereas our equation predicted 2.25/100 sq. km. The difference is a consequence of large-scale human interference as the retaliatory killing of predators, depletion of prey and failure of reintroduced tigers in establish and breed owing to a lot of anthropogenic disturbance in the reserve.

There has been a debate (Kalinkat et al., 2013) regarding the use of linear and curvilinear equations to fit the predator–prey scaling relationship. Karanth et al. (2004) showed the fit from both types of equations and compared the coefficient in the restricted and unrestricted cases. Miquelle et al. (2010) fitted a curvilinear equation to the data of total prey biomass and tiger density from 13 sites. He also tried to fit a linear model to preferred prey biomass and tiger density of nine sites. R-squared values were higher in the case of the curvilinear equation in his case. Hayward, O’Brien & Kerley (2007) used a linear equation for the log-transformed predator and prey densities that are equivalent to using a curvilinear equation. We also used a linear equation with log-transformed ungulate biomass for this model as curvilinear models explain much more variation and provide a more robust fit of the data. This is also ecologically viable as increasing the prey density can only lead to an increasing in predator density till the carrying capacity then reaches the asymptote.

**Figure 3:**
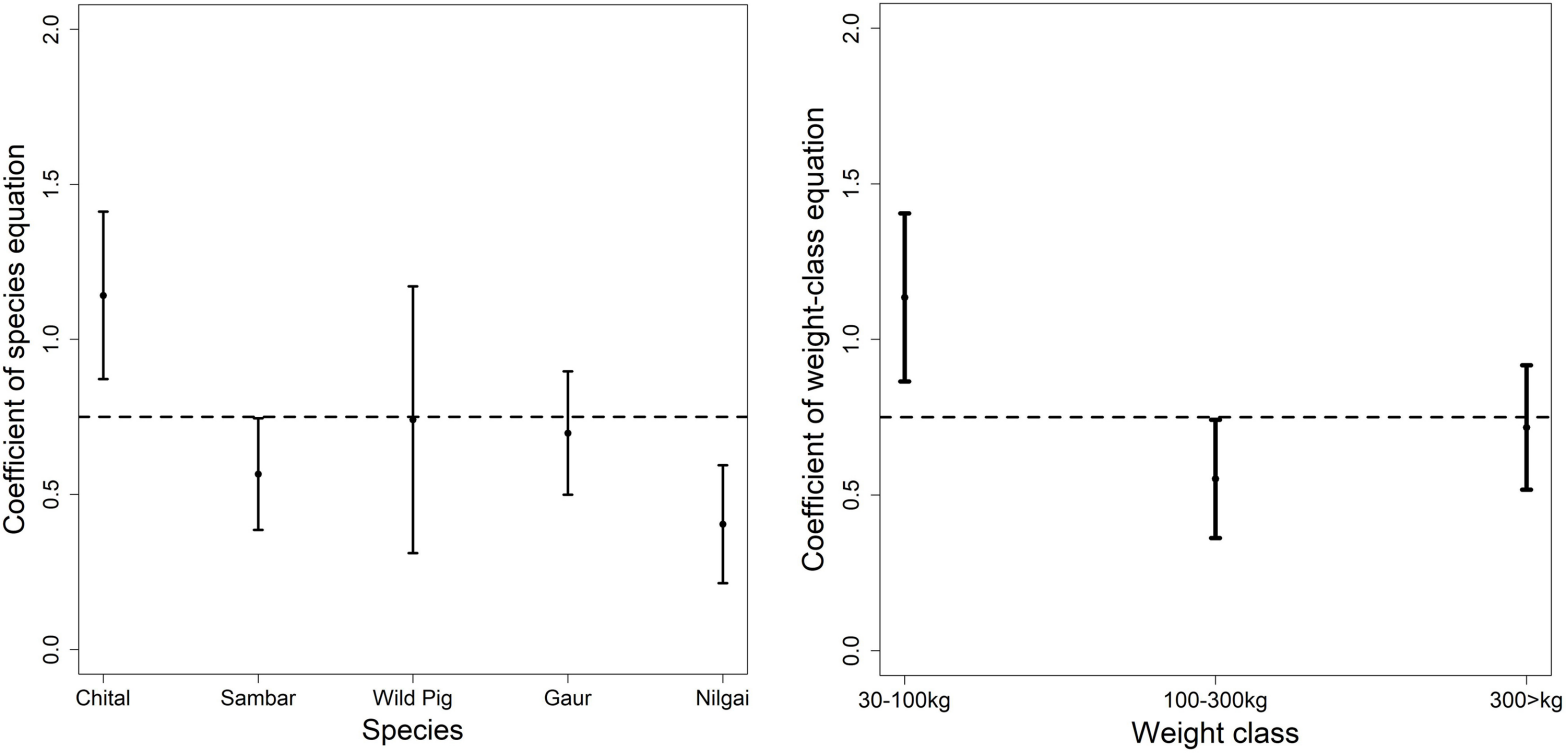
Comparison of the coefficient of the prey species equation and weight equation with 0.75 as reported as the exponent for predator and prey biomass (Hatton et al., 2015).

We compared the coefficients of our predator–prey equations with that of the scaling law given by Hatton et al. (2015). Hatton et al. (2015) analyzed data for tiger, lion and wolf throughout their distribution range to derive equations for large predator and prey species scaling. The coefficient for chital in the prey species equation and medium weight in the weight class equation was greater than 1 (Fig. 3), which shows top-down control of predators on chital but for other prey species, it was mostly controlled bottom-up. Although most of the sites consist of other predators (leopard *Panthera pardus*, dhole *Cuon alpinus etc*.) but evaluating the additive effects would require further evaluation of inter-species interaction. The higher coefficient of chital and medium weight in this scaling equation is concurring with the results of the prey species and weight range equation of this study. Hatton et al. (2015) formulated the equation of tiger and the prey species based on the data majorly from “Project Tiger”. The dataset used in the study (Hatton et al., 2015) is restricted to a few tiger reserves and lacks the analytical rigour (spatial capture-recapture *vs* Individual counts) (Jhala, Qureshi & Gopal, 2015) used in the dataset of the study. Nonetheless, the concurrence of the findings explains the fit of our equation.

## Conclusion

This is the first study to relate the carrying capacity of a large carnivore with relative prey density and weight ranges. The densities were successfully predicted using both the equations and also tested with the available densities. Data collected from diverse habitats show the potential of the equation to be applied to different habitat and productivity gradients. With due potential, long-term population viability for wildlife reserves can be evaluated from the equation that can lead to relocation and reintroduction programmes in future. Substantial deviations from the observed densities can be used to determine the requirements of management interventions in the form of prey species restocking and enhancement or regulation of predator numbers or increased anti-poaching efforts. Conservation actions can also be strengthened by focusing on the status of individual prey species for the long-term survival of both predator and prey species.

##  Supplemental Information

10.7717/peerj.15914/supp-1 Supplemental Information 1 Raw data

10.7717/peerj.15914/supp-2 Supplemental Information 2 Reproducible R codes explaining steps in detail on how the prey density from different tiger reserves was used to derive the tiger carrying capacity equation The text between “‘ “‘ after r are the R codes.

10.7717/peerj.15914/supp-3 Supplemental Information 3 Compiled and Knitted reproducible R codes explaining steps in detail on how the prey density from different tiger reserves was used to derive the tiger carrying capacity equation The highlighted boxes are the R codes.

## Additional Information and Declarations

### Data Availability

The following information was supplied regarding data availability: The raw data is available in the Supplementary File.
