# Supplementary material for: Predicting carrying capacity of a large carnivore from prey densities: a new approach
Source: PeerJ. 2023 Nov 22;11:e15914. doi: 10.7717/peerj.15914 (PMC10676078; doi:10.7717/peerj.15914)
Supplement: Supplemental Information 3 — The highlighted boxes are the R codes. [file peerj-11-15914-s003.pdf]

# Tiger Prey Equation

Nilanjan Chatterjee

15th March, 2023

The `lmtest` and `sandwich` package is used for testing heteroskedasticity in the equation and transform variables to remove heteroskedasticity

```
library(lmtest)
library(sandwich)
library(dplyr)
```

## Load the data and check

```
density_dat <-read.csv("D:/Papers/tiger density India/Submission/Tiger_prey_density_data.csv",header=TRU
```

`log.chital_biomass..chital_biomass_prop`, `log.sambar_biomass..sambar_biomass_prop` these stands for individual species density*proportion of species biomass where,

$$sp_1 biomassproportion = sp_1 density.sp_1 wt / sp_1 density.sp_1 wt + sp_2 density.sp_2 wt + ... + sp_n density.sp_n wt$$

## Data preparation

We calculated individual *species biomass* columns using the log-transformation. We substituted the `NA` values with 0 as $log(0)$ would give the output of `NA`.
Later, we multiplied the *log_species_biomass* column with the *species_biomass_prop* to derive the final columns to be used in the regression equation.

```
density_dat <-density_dat |>
  mutate(log_chital_bio = ifelse(!is.finite(log10(chital_biomass)),0, log10(chital_biomass)),
         log_sambar_bio = ifelse(!is.finite(log10(sambar_biomass)),0, log10(sambar_biomass)),
         log_langur_bio = ifelse(!is.finite(log10(langur_biomass)),0, log10(langur_biomass)),
         log_barkingdeer_bio = ifelse(!is.finite(log10(barking_deer_biomass)),0, log10(barking_deer_bio
         log_wildpig_bio = ifelse(!is.finite(log10(wild_pig_biomass)),0, log10(wild_pig_biomass)),
         log_nilgai_bio = ifelse(!is.finite(log10(nilgai_biomass)),0, log10(nilgai_biomass)),
         log_gaur_bio = ifelse(!is.finite(log10(gaur_biomass)),0, log10(gaur_biomass)))|>
  mutate(chital_bio_prop = log_chital_bio*chital_biomass_prop,
         sambar_bio_prop = log_sambar_bio*sambar_biomass_prop,
         langur_bio_prop = log_langur_bio*langur_biomass_prop,
         gaur_bio_prop = log_gaur_bio*gaur_biomass_prop,
         nilgai_bio_prop = log_nilgai_bio*nilgai_biomass_prop,
         wildpig_bio_prop = log_wildpig_bio*wild_pig_biomass_prop,
         barkingdeer_bio_prop = log_barkingdeer_bio*barking_deer_biomass_prop)

head(density_dat)
```

```
##       Site tiger_density log.tiger_density. chital_biomass sambar_biomass
## 1   Rajaji          2.90          0.4623980          480.6           2412
## 2  Corbett         11.00          1.0413927         1931.4           1818
## 3 Ramnagar          9.71          0.9872192         1152.9            956
## 4 Pilibhit          2.60          0.4149733         1173.9              0
## 5  Valmiki          1.49          0.1731863          119.4            310
## 6                   1.65          0.2174839          567.6           2772
##   langur_biomass barking_deer_biomass wild_pig_biomass nilgai_biomass
## 1          33.85                 0.00              0.0           0.00
## 2         115.90                42.84            522.0         613.47
## 3          39.50                30.52              0.0           0.00
## 4           0.00                 0.00              0.0        1921.53
## 5           0.00                15.96            168.0         748.67
## 6           0.00                 0.00            476.4        2619.50
##   gaur_biomass chital_biomass_prop sambar_biomass_prop langur_biomass_prop
## 1            0          0.16422628           0.8242068          0.01156692
## 2            0          0.38294000           0.3604561          0.02297957
## 3            0          0.52911534           0.4387495          0.01812825
## 4            0          0.37923649           0.0000000          0.00000000
## 5            0          0.08766327           0.2276014          0.00000000
## 6            0          0.08819828           0.4307358          0.00000000
##   barking_deer_biomass_prop wild_pig_biomass_prop nilgai_biomass_prop
## 1               0.000000000            0.00000000           0.0000000
## 2               0.008493916            0.10349730           0.1216331
## 3               0.014006939            0.00000000           0.0000000
## 4               0.000000000            0.00000000           0.6207635
## 5               0.011717804            0.12334530           0.5496722
## 6               0.000000000            0.07402688           0.4070391
##   gaur_biomass_prop log_chital_bio log_sambar_bio log_langur_bio
## 1                 0       2.681784       3.382377       1.529559
## 2                 0       3.285872       3.259594       2.064083
## 3                 0       3.061792       2.980458       1.596597
## 4                 0       3.069631       0.000000       0.000000
## 5                 0       2.077004       2.491362       0.000000
## 6                 0       2.754042       3.442793       0.000000
##   log_barkingdeer_bio log_wildpig_bio log_nilgai_bio log_gaur_bio
## 1            0.000000        0.000000       0.000000            0
## 2            1.631849        2.717671       2.787793            0
## 3            1.484585        0.000000       0.000000            0
## 4            0.000000        0.000000       3.283647            0
## 5            1.203033        2.225309       2.874290            0
## 6            0.000000        2.677972       3.418218            0
##   chital_bio_prop sambar_bio_prop langur_bio_prop gaur_bio_prop nilgai_bio_prop
## 1       0.4404194       2.7877784      0.01769228             0        0.000000
## 2       1.2582919       1.1749405      0.04743175             0        0.339088
## 3       1.6200409       1.3076743      0.02894351             0        0.000000
## 4       1.1641161       0.0000000      0.00000000             0        2.038368
## 5       0.1820770       0.5670375      0.00000000             0        1.579917
## 6       0.2429018       1.4829342      0.00000000             0        1.391348
##   wildpig_bio_prop barkingdeer_bio_prop
## 1        0.0000000           0.00000000
## 2        0.2812716           0.01386079
## 3        0.0000000           0.02079448
## 4        0.0000000           0.00000000
```

```
## 5        0.2744814            0.01409690
## 6        0.1982419            0.00000000
```

## Regression

Ordinary linear regression equation for species-wise prey-predator equation

```
aleqt1 <-lm(log.tiger_density. ~ chital_bio_prop + sambar_bio_prop +
                                 langur_bio_prop + gaur_bio_prop +  nilgai_bio_prop +
                                 wildpig_bio_prop + barkingdeer_bio_prop, data = density_dat)
```

### Checking for heteroskedasticity

Conducting Breusch-Pagan test for heteroskedasticity and estimating the heteroskedasticity-consistent covariane matrix. Comapring the standard error of the equation with the robust standard errors reflect which variable has the major issue with variance. There are multiple options for the covariance matrix, this HC4m was recommended by Cribari-Neto & Da Silva 2011.

```
summary(aleqt1)
```

```
##
## Call:
## lm(formula = log.tiger_density. ~ chital_bio_prop + sambar_bio_prop +
##     langur_bio_prop + gaur_bio_prop + nilgai_bio_prop + wildpig_bio_prop +
##     barkingdeer_bio_prop, data = density_dat)
##
## Residuals:
##      Min       1Q   Median       3Q      Max
## -0.74458 -0.13151  0.01724  0.20239  0.41611
##
## Coefficients:
##                      Estimate Std. Error t value Pr(>|t|)
## (Intercept)           -1.6133     0.6925  -2.330  0.03419 *
## chital_bio_prop        1.2102     0.3169   3.819  0.00168 **
## sambar_bio_prop        0.6727     0.2320   2.899  0.01102 *
## langur_bio_prop       -2.6442     2.3953  -1.104  0.28703
## gaur_bio_prop          0.7411     0.2240   3.309  0.00477 **
## nilgai_bio_prop        0.4068     0.2296   1.772  0.09678 .
## wildpig_bio_prop       1.0853     0.5538   1.960  0.06889 .
## barkingdeer_bio_prop  -9.5009     5.3835  -1.765  0.09794 .
## ---
## Signif. codes:  0 '***' 0.001 '**' 0.01 '*' 0.05 '.' 0.1 ' ' 1
##
## Residual standard error: 0.343 on 15 degrees of freedom
## Multiple R-squared:  0.6291, Adjusted R-squared:  0.456
## F-statistic: 3.635 on 7 and 15 DF,  p-value: 0.01709
```

```
coeftest(aleqt1, vcov = vcovHC(aleqt1, "HC4m"))
```

```
##
## t test of coefficients:
```

```
##
##                        Estimate Std. Error t value Pr(>|t|)
## (Intercept)           -1.61334    1.07820 -1.4963  0.15531
## chital_bio_prop        1.21019    0.49851  2.4276  0.02825 *
## sambar_bio_prop        0.67268    0.35920  1.8727  0.08073 .
## langur_bio_prop       -2.64421    2.42735 -1.0893  0.29320
## gaur_bio_prop          0.74107    0.31313  2.3667  0.03183 *
## nilgai_bio_prop        0.40682    0.35019  1.1617  0.26351
## wildpig_bio_prop       1.08526    0.51221  2.1188  0.05121 .
## barkingdeer_bio_prop -9.50086    6.63240 -1.4325  0.17251
## ---
## Signif. codes:  0 '***' 0.001 '**' 0.01 '*' 0.05 '.' 0.1 ' ' 1
```

```
bptest(aleqt1)
```

```
##
##  studentized Breusch-Pagan test
##
## data:  aleqt1
## BP = 8.5713, df = 7, p-value = 0.2849
```

### Plotting

Plotting the residuals vs Fitted and the modulus of the residuals for each of the explanatory variable. We detected a decrease in the residuals of the chital variable as the value increases. This was supposed to be a source of heteroskedascity and we did transformation to remove that

```
attach(density_dat)
par(mfrow=c(3,2))
par(mar=c(4,4,1,1))
plot(aleqt1$fitted.values, aleqt1$residuals, xlab="fitted", ylab="residuals", pch=19)
plot(chital_bio_prop, abs(aleqt1$residuals), xlab="Chital", ylab="residuals", pch=19)
plot(sambar_bio_prop, abs(aleqt1$residuals), xlab="Sambar", ylab="residuals", pch=19)
plot(wildpig_bio_prop, abs(aleqt1$residuals), xlab="Wild-pig", ylab="residuals", pch=19)
plot(nilgai_bio_prop, abs(aleqt1$residuals), xlab="Nilgai", ylab="residuals", pch=19)
plot(gaur_bio_prop, abs(aleqt1$residuals), xlab="Gaur", ylab="residuals", pch=19)
```

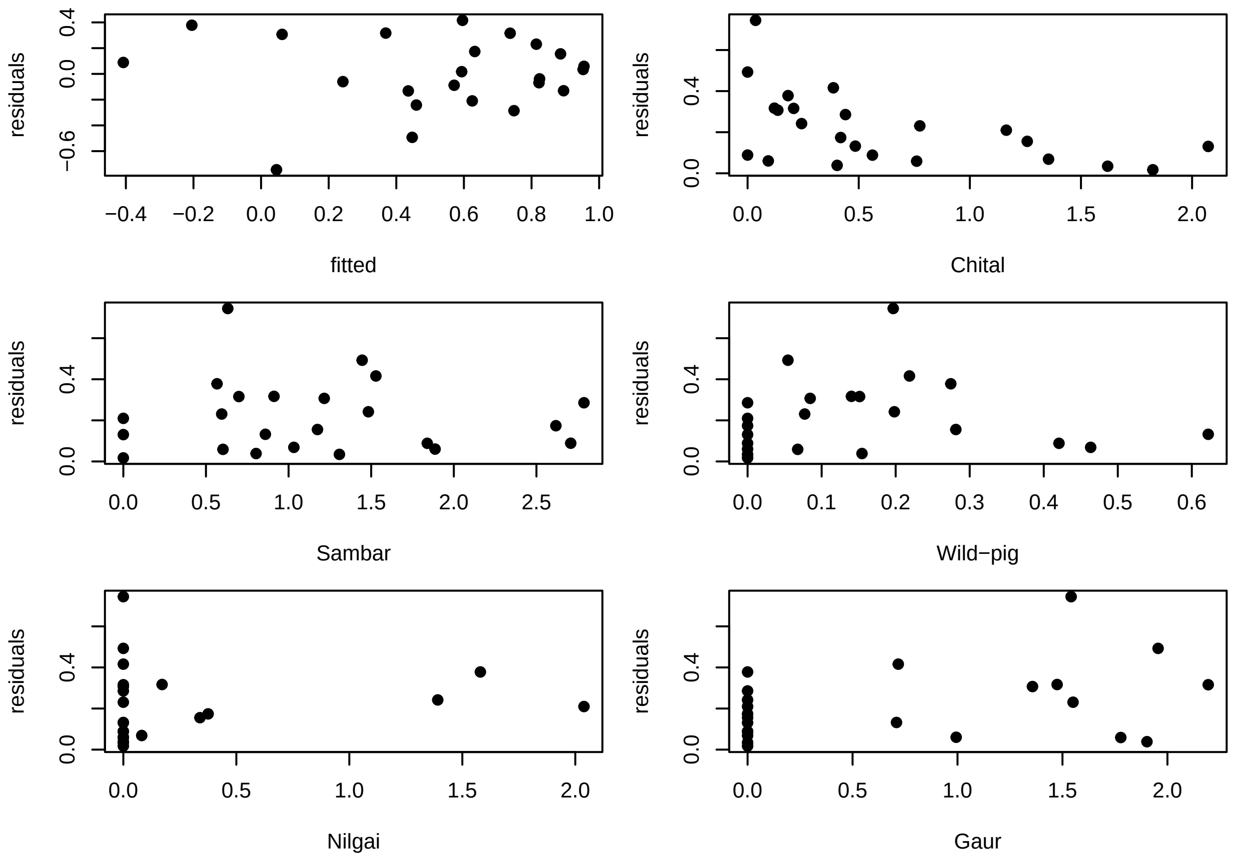


## Transformation

We did one transformation for converting the heteroskedastic model into a homoskedastic one

$$\sigma_i^2 = \sigma^2/((x_i + 1)$$

We multiplied the previous equation with

$$\sqrt(x_i + 1)$$

for the transformation

### First transformation

We used a square-root transformation first to remove the heteroskedasticity from the variables.

```
x1 <-sqrt(chital_bio_prop+1)
y11 <-log.tiger_density.*x1
x2 <- chital_bio_prop*x1
x3 <- sambar_bio_prop*x1
x4 <- wildpig_bio_prop*x1
x5 <- nilgai_bio_prop*x1
x6 <- gaur_bio_prop*x1
x7 <- langur_bio_prop*x1
x8 <- barkingdeer_bio_prop*x1
```

#### New linear regression equation with the transformed variable

```
new_eqn <-lm (y11 ~x1 +x2+x3+x4+x5+x6-1)
summary(new_eqn)
```

```
##
## Call:
## lm(formula = y11 ~ x1 + x2 + x3 + x4 + x5 + x6 - 1)
##
## Residuals:
##      Min       1Q   Median       3Q      Max
## -0.78452 -0.16000 -0.02785  0.25200  0.69635
##
## Coefficients:
##    Estimate Std. Error t value Pr(>|t|)
## x1  -1.5491     0.5809  -2.667 0.016267 *
## x2   1.1419     0.2686   4.251 0.000539 ***
## x3   0.5664     0.1825   3.104 0.006449 **
## x4   0.7413     0.4255   1.742 0.099509 .
## x5   0.4035     0.1900   2.124 0.048637 *
## x6   0.6981     0.1992   3.504 0.002721 **
## ---
## Signif. codes:  0 '***' 0.001 '**' 0.01 '*' 0.05 '.' 0.1 ' ' 1
##
## Residual standard error: 0.3954 on 17 degrees of freedom
## Multiple R-squared:  0.8677, Adjusted R-squared:  0.821
## F-statistic: 18.58 on 6 and 17 DF,  p-value: 1.325e-06
```

```
bptest(new_eqn)
```

```
##
##  studentized Breusch-Pagan test
##
## data:  new_eqn
## BP = 4.5292, df = 5, p-value = 0.476
```

#### Plotting

The residual vs fitted plot and the species wise residuals looks like the following. Here the residuals have significantly less heteroskedasticity compared to the the earlier equation.

```
par(mfrow=c(3,2))
par(mar=c(4,4,1,1))
plot(new_eqn$fitted.values, new_eqn$residuals, xlab="fitted", ylab="residuals", pch=19)
plot(x2, abs(new_eqn$residuals), xlab="Chital", ylab="residuals", pch=19)
plot(x3, abs(new_eqn$residuals), xlab="Sambar", ylab="residuals", pch=19)
plot(x4, abs(new_eqn$residuals), xlab="Wild-pig", ylab="residuals", pch=19)
plot(x5, abs(new_eqn$residuals), xlab="Nilgai", ylab="residuals", pch=19)
plot(x6, abs(new_eqn$residuals), xlab="Gaur", ylab="residuals", pch=19)
```

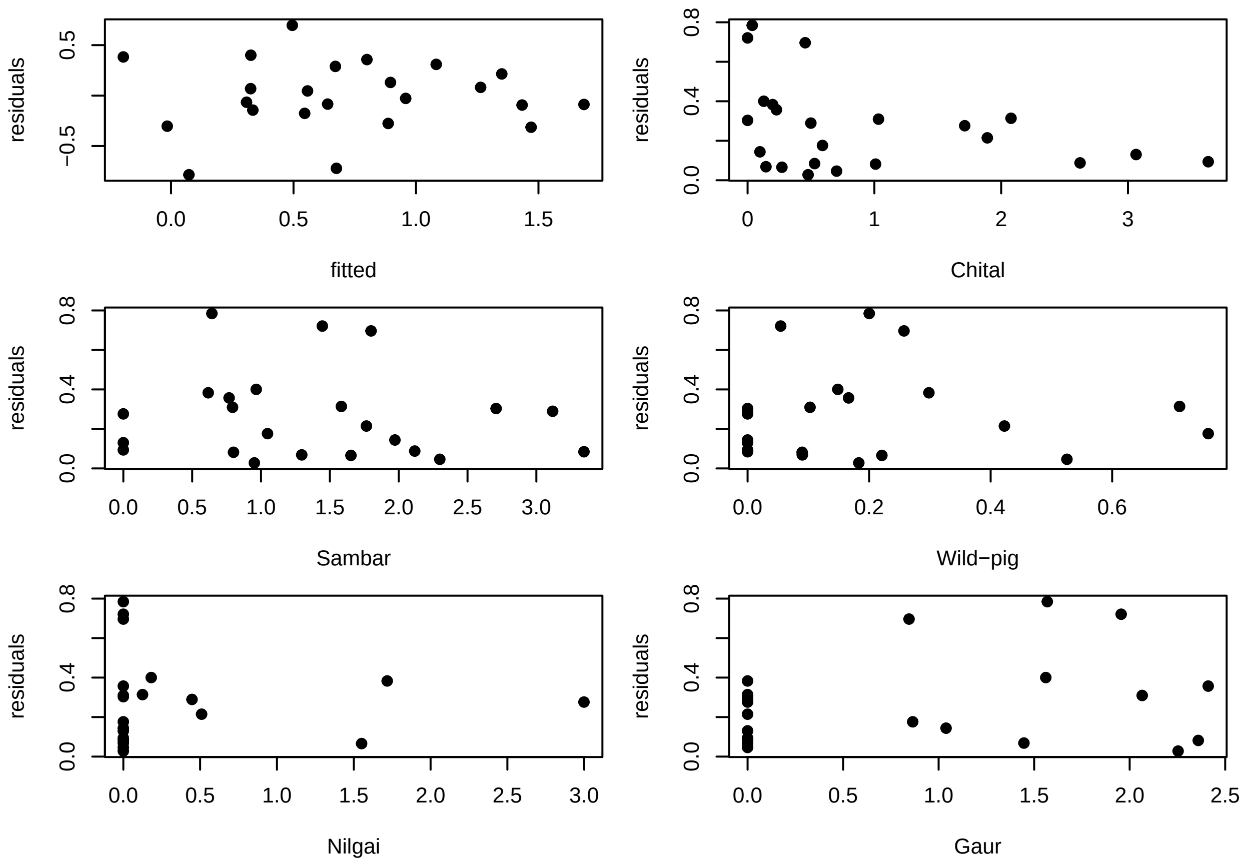
residuals
fitted
Chital
Sambar
Wild−pig
Nilgai
Gaur
